# Molecular cloning and characterization of a grapevine (*Vitis vinifera* L.) serotonin *N*-acetyltransferase (*VvSNAT2*) gene involved in plant defense

**DOI:** 10.1186/s12864-019-6085-3

**Published:** 2019-11-20

**Authors:** Yihe Yu, Lu Bian, Zeling Jiao, Keke Yu, Yutong Wan, Guohai Zhang, Dalong Guo

**Affiliations:** Henan Engineering Technology Research Center of Quality Regulation and Controlling of Horticultural Plants, College of Forestry, Henan University of Science and Technology, Luoyang, Henan Province 471023 People’s Republic of China

**Keywords:** Melatonin, *Vitis vinifera*, Serotonin *N*-acetyltransferase, *VvSNAT2*, Defense

## Abstract

**Background:**

Melatonin is a ubiquitous molecule and exists across kingdoms. Studies on melatonin in plants have mainly focused on its physiological influence on growth and development, and on its biosynthesis. A number of studies have been conducted on the melatonin content and exogenous melatonin treatment of grapevine (*Vitis vinifera* L.). However, key genes or enzymes of the melatonin biosynthetic pathway remain unclear.

**Results:**

In this study, we cloned and identified the gene encoding serotonin *N*-acetyltransferase (SNAT) in grapevine (*VvSNAT2*)*.* The VvSNAT2 protein was identified from a collection of 30 members of the grapevine GCN5-related *N*-acetyltransferase (GNAT) superfamily. Phylogenetic and protein sublocalization analyses showed that the candidate gene *VvGNAT16* is *VvSNAT2*. Characterization of VvSNAT2 showed that its enzymatic activity is highest at a pH of 8.8 and a temperature of 45 °C. Analysis of enzyme kinetics showed the values of *K*_m_ and *V*_max_ of VvSNAT2 using serotonin were 392.5 μM and 836 pmol/min/mg protein, respectively. The expression of VvSNAT2 was induced by melatonin treatment and pathogen inoculation. Overexpression of *VvSNAT2* in Arabidopsis resulted in greater accumulation of melatonin and chlorophyll and enhanced resistance to powdery mildew in the transgenic plants compared with the wild type (WT). Additionally, our data showed that the marker genes in the salicylic acid (SA) signaling pathway were expressed to higher levels in the transgenic plants compared with the WT.

**Conclusions:**

The *VvSNAT2* gene was cloned and identified in grapevine for the first time. Our results indicate that *VvSNAT2* overexpression activates the SA and JA signaling pathways; however, the SA pathway plays a central role in *VvSNAT2*-mediated plant defense.

## Highlight

*VvSNAT2* was identified in grapevine, which mediated SA signaling pathway plays a central role in disease resistance.

## Background

Melatonin (*N*-acetyl-5-methoxytryptamine) was originally identified in and isolated from the pineal gland of cows [1]. Melatonin has an indole-based structure and is the most versatile and ubiquitous hormone in living organisms, including macroalgae, bacteria, fungi, plants, animals and humans [2]. Melatonin performs many important functions in animals and humans, such as maintaining circadian rhythmicity, delaying aging, preventing or reversing cancer, facilitating seasonal reproduction and enhancing innate immune responses [2–4]. Since its discovery in plants, melatonin has been shown to play a key role in seedling growth, flower and fruit development, leaf senescence, photosynthesis and biotic and abiotic stress [5–7].

Melatonin is synthesized from L-tryptophan by the consecutive actions of four enzymes, including tryptophan decarboxylase (TDC), tryptamine5-hydroxylase (T5H), serotonin *N*-acetyltransferase (SNAT) and *N*-acetylserotonin methyltransferase (ASMT) [4]. The *SNAT* gene has been cloned and functionally characterized in several plants species. The rice (*Oryza sativa* L.) genome harbors two copies of *SNAT*, including *OsSNAT1* and *OsSNAT2* [8, 9]. Both these genes belong to the GCN5-related *N*-acetyltransferase (GNAT) superfamily; the GNAT proteins share 39% sequence identity and 60% sequence similarity [8, 9]. Both OsSNAT1 and OsSNAT2 show SNAT enzymatic activity in *Escherichia coli* and in vitro*,* thus producing *N*-acetyltryptamine [8, 9]. Rice plants overexpressing *OsSNAT1* exhibit increased melatonin levels, resistance to cadmium toxicity and delayed senescence [10]. Additionally, the T2 homozygous plants overexpressing *OsSNAT1* exhibit higher grain yield because of increased panicle number per plant under paddy field conditions [10]. The amino acid sequence of SNAT in cyanobacterium (cSNAT) shows 56% homology with OsSNAT1 [11]. The purified cSNAT protein exhibits SNAT enzymatic activity, especially under high temperature [11]. In loblolly pine, SNAT has been shown to localize in chloroplasts [12]. Purified recombinant PtSNAT protein shows SNAT enzymatic activity [12]. The *SNAT* gene has also been cloned in *Pyropia yezoensis*; PySNAT localizes to the cytoplasm because it lacks N-terminal chloroplast transit peptides [12]. Compared with animals, only a few *SNAT* genes have been cloned in plants.

Grapevine (*Vitis vinifera* L.) is one of the most widely cultivated fruit trees in the world [13, 14]. Grapes are considered as a health-promoting fruit because they not only contain a high level of resveratrol but also produce melatonin [15, 16]. The melatonin content of grapes varies with the cultivar, organ and developmental stage. The melatonin content of berry skin ranges from 0.005 to 0.965 ng/g among eight different cultivars [17]. Berry skin of the Argentinian cultivar ‘Malbec’ contains a much higher concentration of melatonin (9.3–17.5 ng/g) than other cultivars [18]. In grape berries pre-veraison, melatonin content is the highest in the skin compared with that in seeds and flesh [19]. During veraison, the skin melatonin content decreases by 47%; however, the melatonin content of seeds and flesh increases by 63 and 95%, respectively, after veraison [20]. Melatonin levels in grape berries are also affected by the circadian rhythym in grapevines grown under field conditions [19]. Although considerable research has been conducted on the melatonin content and health-related functions of grapevine, key enzymes involved in the melatonin biosynthesis pathway have not yet been identified.

In this study, we cloned a member of the grapevine *GNAT* gene family, *VvSNAT2*, and identified its function. We expressed *VvSNAT2* in *E. coli* and purified the VvSNAT2-His fusion protein to characterize its enzymatic activity. Phylogenetic analysis, enzyme activity characterization and protein localization analysis showed that VvGNAT16 is VvSNAT2. Protein expression indicated that VvSNAT2 was induced by melatonin treatment and pathogen inoculation. Furthermore, transgenic Arabidopsis overexpressing *VvSNAT2* revealed its role in plant defense against pathogens.

## Results

### Candidate VvSNAT screen and sequence analysis

**Fig. 1:**
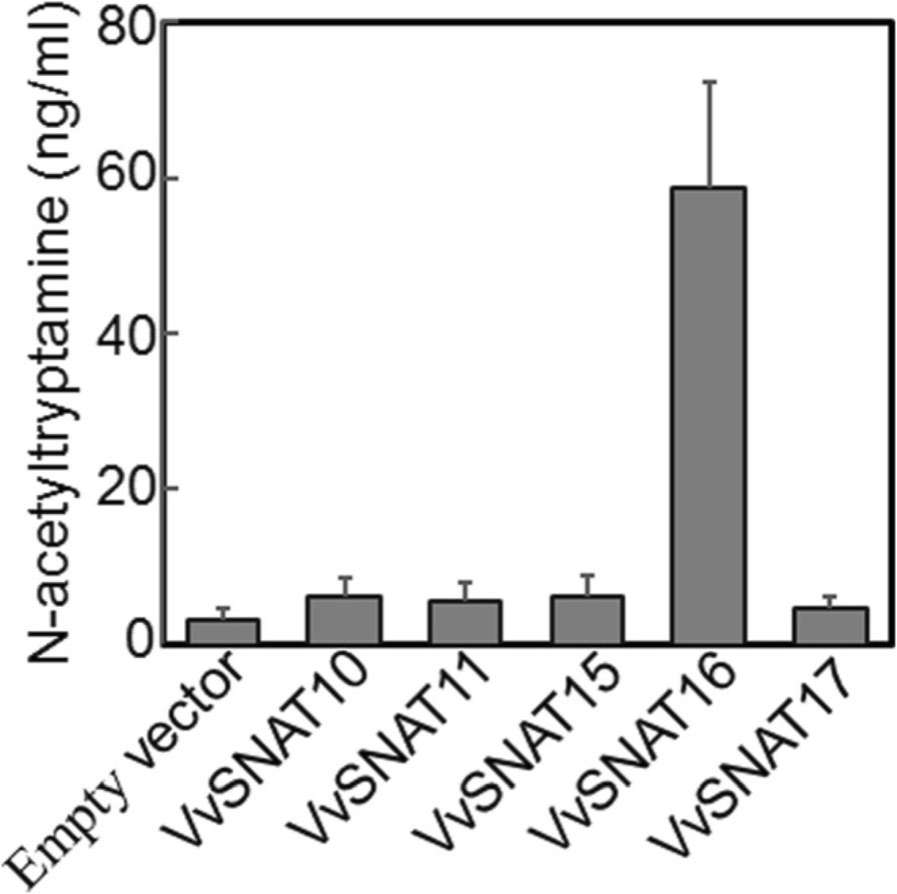
N-acetyltryptamine production in *Escherichia coli. VvGNAT genes* and empty vector were transformed into *E. coli.* The expression of VvGNAT proteins was induced by the addition of IPTG, and enzyme activity was measured in the presence of 1 mM tryptamine for 12 h. The bacterial pellets were collected and used for the quantification of *N*-acetyltryptamine using HPLC. Data represent mean ± standard deviation (SD) of triplicate experiments

**Fig. 2:**
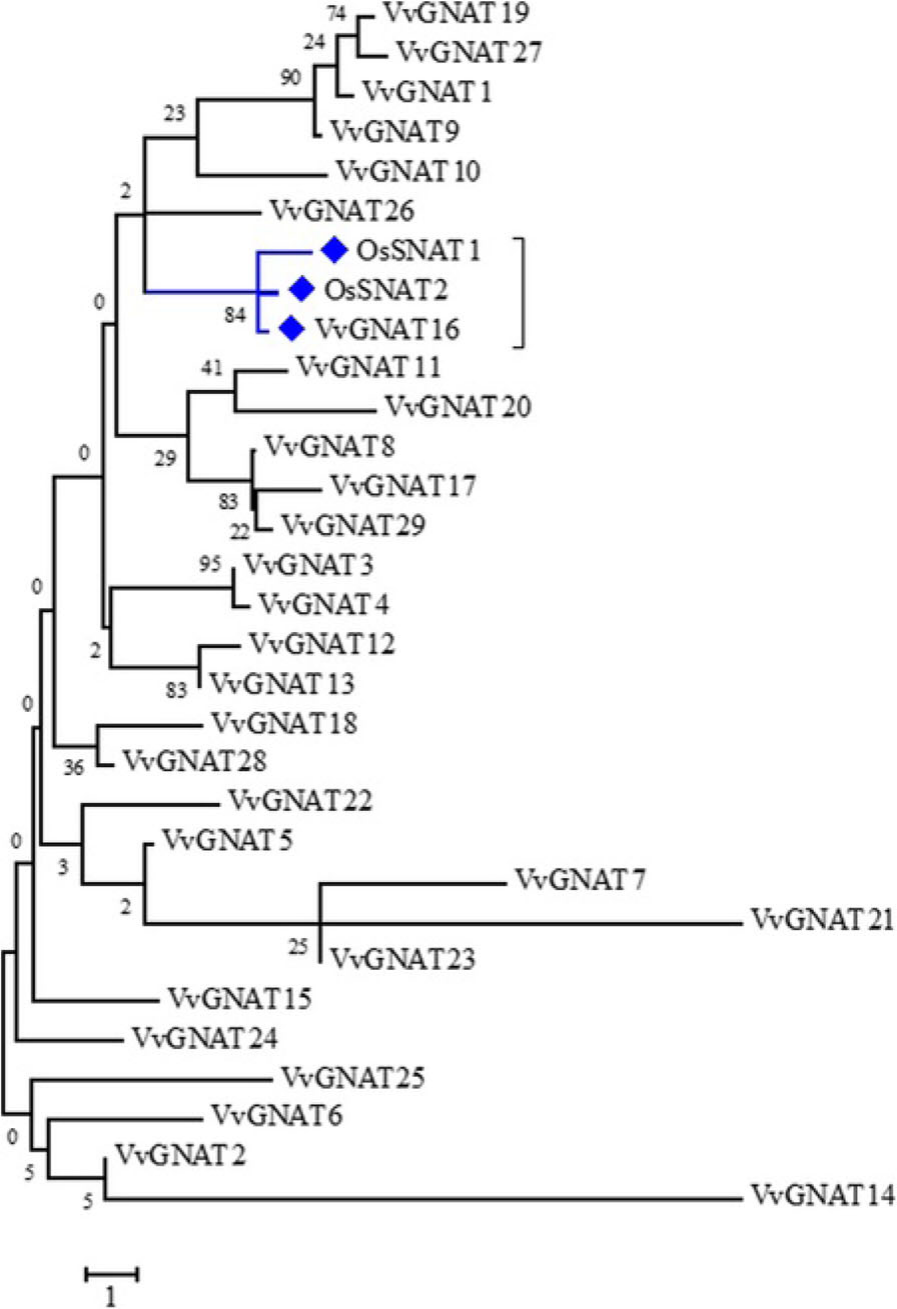
Phylogenetic analysis of grapevine GNAT superfamily and rice SNAT proteins. Multiple sequence alignment of grapevine GNAT and rice SNAT proteins was performed, and a phylogenetic tree was constructed with the MEGA-X software using the neighbor-joining method. Bootstrap values of 1000 replicates are shown in percentages at the branch nodes. OsSNAT1, XP_015637887; OsSNAT2, XP_015648698

Nucleotide sequences of 30 grapevine *GNAT* superfamily (Pfam00583) genes (Table 1) were downloaded from the grapevine genome database. Chloroplast transit signal peptide was identified in five sequences, including *VvGNAT10*, *VvGNAT11*, *VvGNAT15*, *VvGNAT16* and *VvGNAT17* (Table 1). Coding sequences of these five genes minus the N-terminal chloroplast transit signal peptide were cloned into the expression vector and expressed in *E. coli*. The SNAT enzyme uses tryptamine as a substrate to synthesis *N*-acetyltryptamine.^9^ To determine whether these five sequences produced *N*-acetyltryptamine in the presence of tryptamine, transgenic *E. coli* expressing these five genes were cultured independently. After tryptamine induction for 12 h, the cell pellet of each transgenic *E. coli* was analyzed by HPLC. Results showed that *E. coli* expressing *VvGNAT16* produced *N*-acetyltryptamine to higher levels than those expressing the other four genes (Fig. 1). Phylogenetic analysis of VvGNAT16 with OsSNAT1 and OsSNAT2 [8, 11] showed that all three proteins grouped into the same subfamily with a high sequence identity with OsSNAT2 (55%) (Fig. 2), indicating that VvGNAT16 cDNA encodes the grapevine SNAT2 protein. Therefore, we renamed *VvGNAT16* as *VvSNAT2*. Sequence analysis showed that the open reading frame (ORF) of *VvSNAT2* is 549 bp, which encodes a polypeptide containing 182 amino acid residues with a predicted molecular mass of 20.2 kDa. The chloroplast transit signal peptide was located at the N-terminal end of VvSNAT2. BLAST analysis revealed VvSNAT2 protein homologs in various plant species, such as *Populus trichocarpa* (77%), *Malus domestica* (73%), *Prunus mume* (73%), *Prunus persica* (71%) and *Citrus sinensis* (74%), OsSNAT2 (55%) and OsSNAT1 (41%).

**Table 1:** List of the gene information of grapevine GCN5-related N-acetyltransferases (GNAT) superfamily

Gene	Accession no.	aa length	Chr. locus	Chloroplast transit peptide
VvGNAT1	VIT_07s0129g00260	421	Chr7	No
VvGNAT2	VIT_17s0000g00600	309	Unknown	No
VvGNAT3	VIT_07s0141g00140	416	Chr7	No
VvGNAT4	VIT_07s0141g00150	438	Chr7	No
VvGNAT5	VIT_07s0151g01030	174	Chr7	No
VvGNAT6	VIT_07s0151g00510	127	Chr7	No
VvGNAT7	VIT_17s0000g10190	455	Chr17	No
VvGNAT8	VIT_11s0016g01160	523	Chr11	No
VvGNAT9	VIT_09s0018g00300	400	Chr9	No
VvGNAT10	VIT_13s0019g04570	220	Chr13	Yes
VvGNAT11	VIT_13s0019g04360	270	Chr13	Yes
VvGNAT12	VIT_05s0020g03680	172	Chr5	No
VvGNAT13	VIT_05s0020g03690	180	Chr5	No
VvGNAT14	VIT_01s0026g00590	249	Chr1	No
VvGNAT15	VIT_07s0151g01010	125	Chr7	Yes
VvGNAT16	VIT_01s0010g01140	182	Chr1	Yes
VvGNAT17	VIT_11s0037g01280	279	Chr11	Yes
VvGNAT18	VIT_16s0039g01810	254	Chr16	No
VvGNAT19	VIT_06s0004g06950	384	Chr6	No
VvGNAT20	VIT_18s0041g01220	287	Chr18	No
VvGNAT21	VIT_12s0057g00440	195	Chr12	No
VvGNAT22	VIT_12s0059g00170	158	Chr12	No
VvGNAT23	VIT_13s0064g00020	160	Chr13	No
VvGNAT24	VIT_14s0068g01050	204	Chr14	No
VvGNAT25	VIT_08s0007g05900	666	Chr8	No
VvGNAT26	VIT_08s0007g03320	157	Chr8	No
VvGNAT27	VIT_05s0077g01020	417	Chr5	No
VvGNAT28	VIT_04s0008g04740	164	Chr4	No
VvGNAT29	VIT_06s0009g01940	288	Chr6	No

### Characterization of VvSNAT2 enzymatic activity

**Fig. 3:**
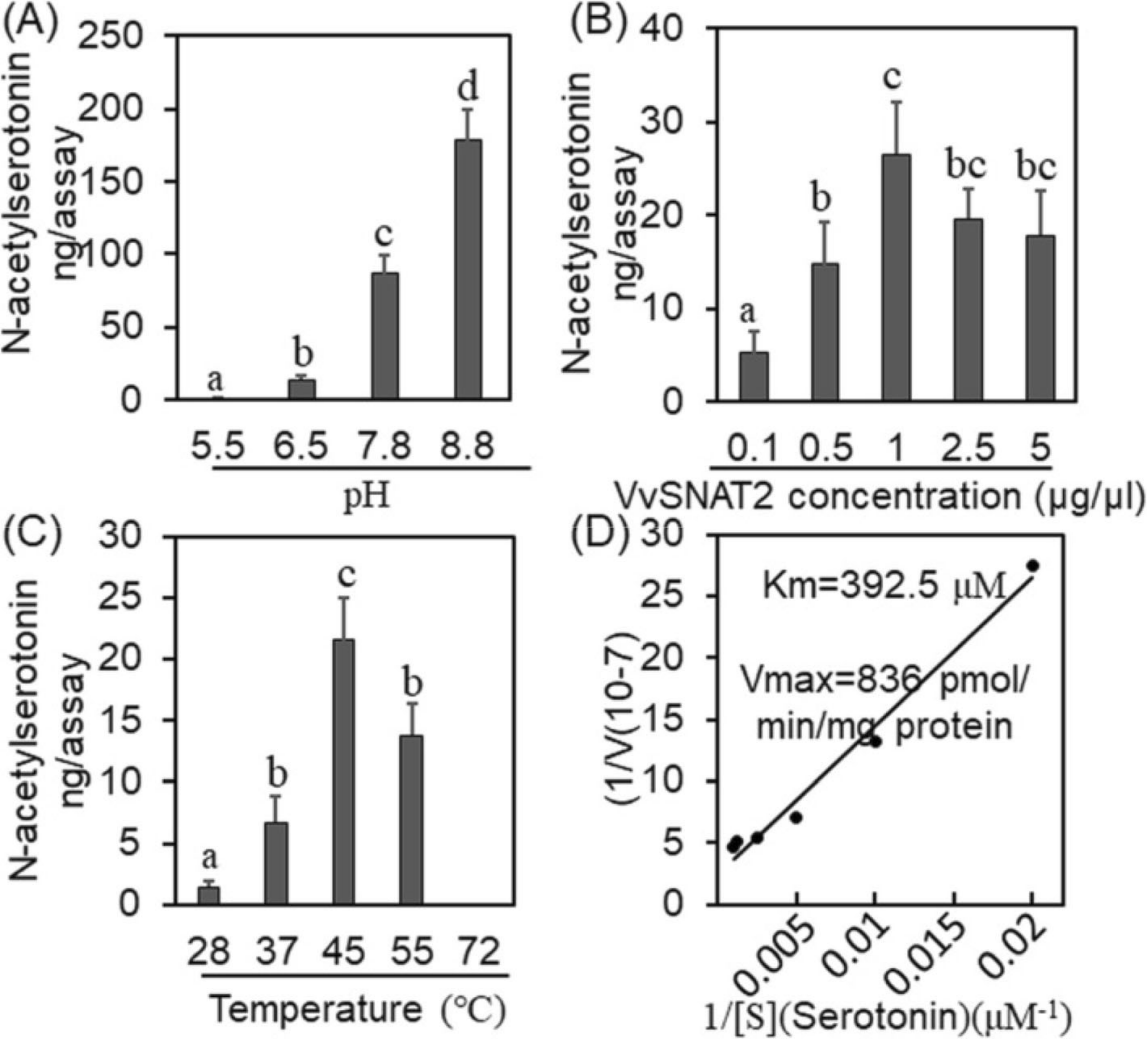
Characterization of enzymatic activity of VvSNAT2. **a–c** Analysis of the enzymatic activity of VvSNAT2 at different pH (**a**), protein concentration (**b**) and temperature (**c**). **d** Determination of *K*_m_ and *V*_max_ values of VvSNAT2 using serotonin as a substrate. VvSNAT2 (1 μg) was incubated with variable substrate concentrations for 30 min at 45 °C. The expression of VvSNAT2 protein in transformed bacteria was induced by the addition of IPTG and 1 mM tryptamine for 12 h. The bacterial pellets were collected and used for the quantification of *N-*acetyltryptamine via HPLC. The *K*_m_ and *V*_max_ values were determined using Lineweaver–Burk plots. Data represent mean ± SD of triplicate experiments. Different letters indicate statistically significant differences at *P* < 0.05

To characterize the enzymatic activity of VvSNAT2, a C-terminal histidine-tag fusion of VvSNAT2 was expressed in *E. coli* and detected by SDS-PAGE (Additional file 1: Figure S1). After affinity purification, the purified VvSNAT2-HIS fusion protein was examined by SDS-PAGE and used to measure VvSNAT2 enzymatic activity and kinetics in vitro. The enzymatic activity of VvSNAT2 was detected at a pH of 6.5 and continued to increase with increasing pH, reaching a peak at pH 8.8 (Fig. 3a). VvSNAT2 exhibited the highest enzymatic activity at a concentration of 1 μg/ml (Fig. 3b). The reaction temperature also affected the enzymatic activity of VvSNAT2; VvSNAT2 showed peak activity at 45 °C and no activity at 72 °C (Fig. 3c). The values for *K*_m_ and *V*_max_ using serotonin were 392.5 μM and 836 pmol/min/mg protein, respectively (Fig. 3d).

### Sublocalization of VvSNAT2

**Fig. 4:**
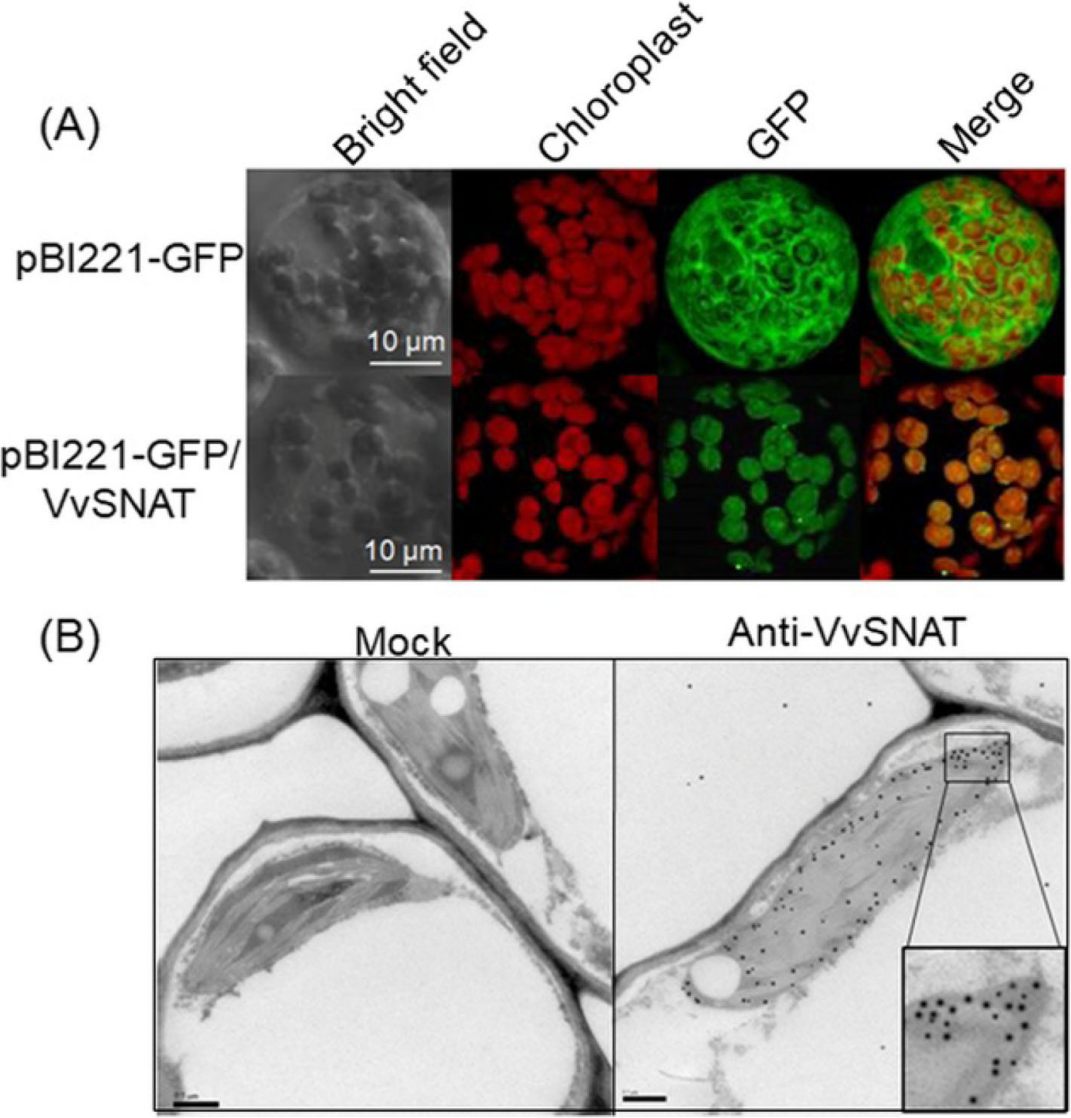
Sublocalization of VvSNAT2. **a** Transient expression of VvSNAT2-GFP in Arabidopsis protoplasts. pBI221-GFP/VvSNAT2 and control pBI221-GFP plasmids were introduced into Arabidopsis protoplasts via PEG-meditated transformation. The transformed protoplasts were incubated in the dark for 14 h and then visualized using confocal microscopy. **b** Immunogold labeling of VvSNAT2 using ultrathin sections of grapevine leaves. Sections were incubated with antiserum diluted 100-fold in 1% (w/v) BSA in TBS buffer for 1 h. After washing the samples with TBS buffer, gold-labeled sections were examined under a transmission electron microscope. Rabbit pre-immune serum was used as a control

To determine whether VvSNAT2 localized to chloroplasts, the *VvSNAT2-GFP* fusion construct was transiently expressed in Arabidopsis protoplasts, and GFP signal was analyzed using confocal microscopy. As shown in Fig. 4a, the control plasmid pBI221-GFP showed GFP signal in the entire protoplast, whereas the VvSNAT2-GFP showed GFP signal only in the chloroplast (Fig. 4a). These results were further confirmed in vivo using immunogold analyses of ultrathin sections of grapevine leaves. Gold particles were abundant in the chloroplast (Fig. 4b). Although gold labeling was also observed in the cytoplasm, its abundance was much lower (Fig. 4b). In control experiments, ultrathin sections incubated with the pre-immune serum showed no gold labeling (Fig. 4b).

### Expression analysis of VvSNAT2 protein

**Fig. 5:**
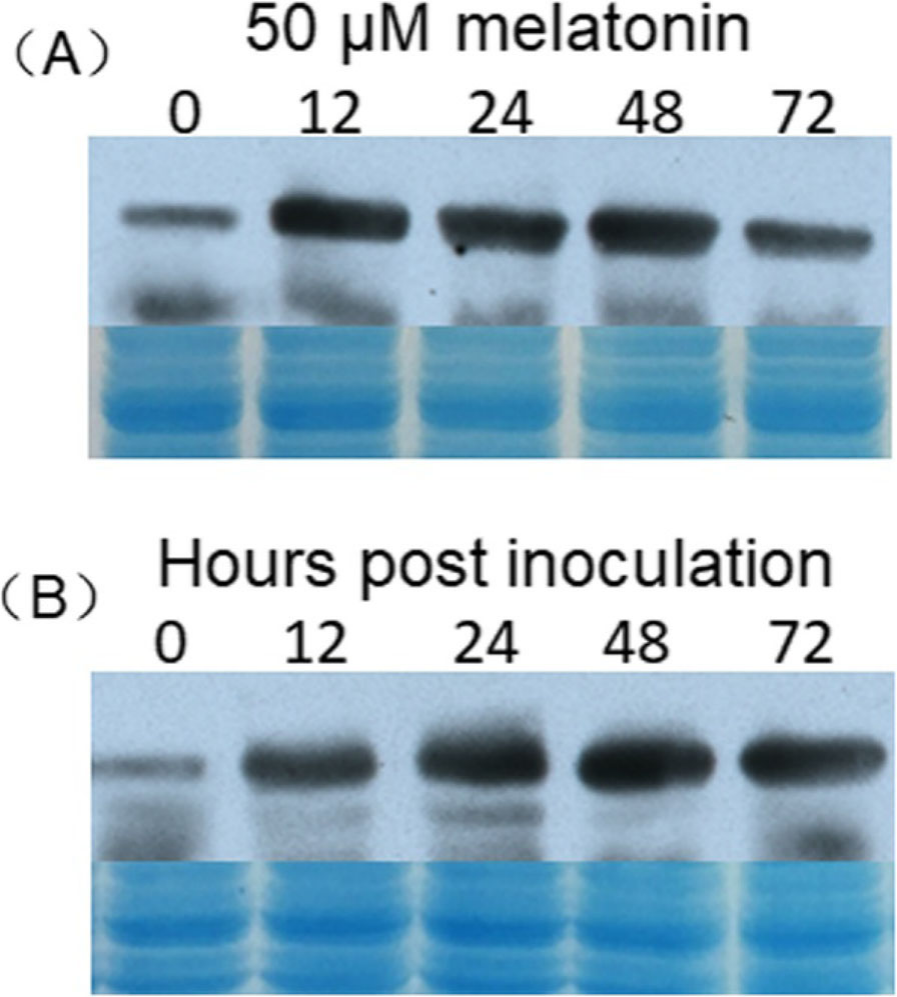
Western blot analysis of VvSNAT2 in response to melatonin treatment and pathogen inoculation. **a** and **b** VvSNAT2 protein accumulation in response to melatonin treatment (**a**) and pathogen inoculation (**b**). Protein extracted from grapevine leaves was quantified using the Bradford assay, and of 20 μg total protein was loaded on the gel for PAGE. Coomassie brilliant blue stained gel was used as a loading control

To examine VvSNAT2 protein expression in response to melatonin treatment and powdery mildew challenge, we preformed western blot analysis using anti-VvSNAT2 serum. After melatonin treatment 12 h, VvSNAT2 quickly accumulated to approximately 8.5-fold higher level than that at 0 h (Fig. 5a). The accumulation of VvSNAT2 was the highest at 48 h and then decreased by 60 h (Fig. 5a). Pathogen inoculation induced the expression of VvSNAT2, with the highest protein accumulation at 48 h post-inoculation (Fig. 5b). These results showed that VvSNAT2 was induced by melatonin treatment and pathogen infection.

### Overexpression of *VvSNAT2* in Arabidopsis

**Fig. 6:**
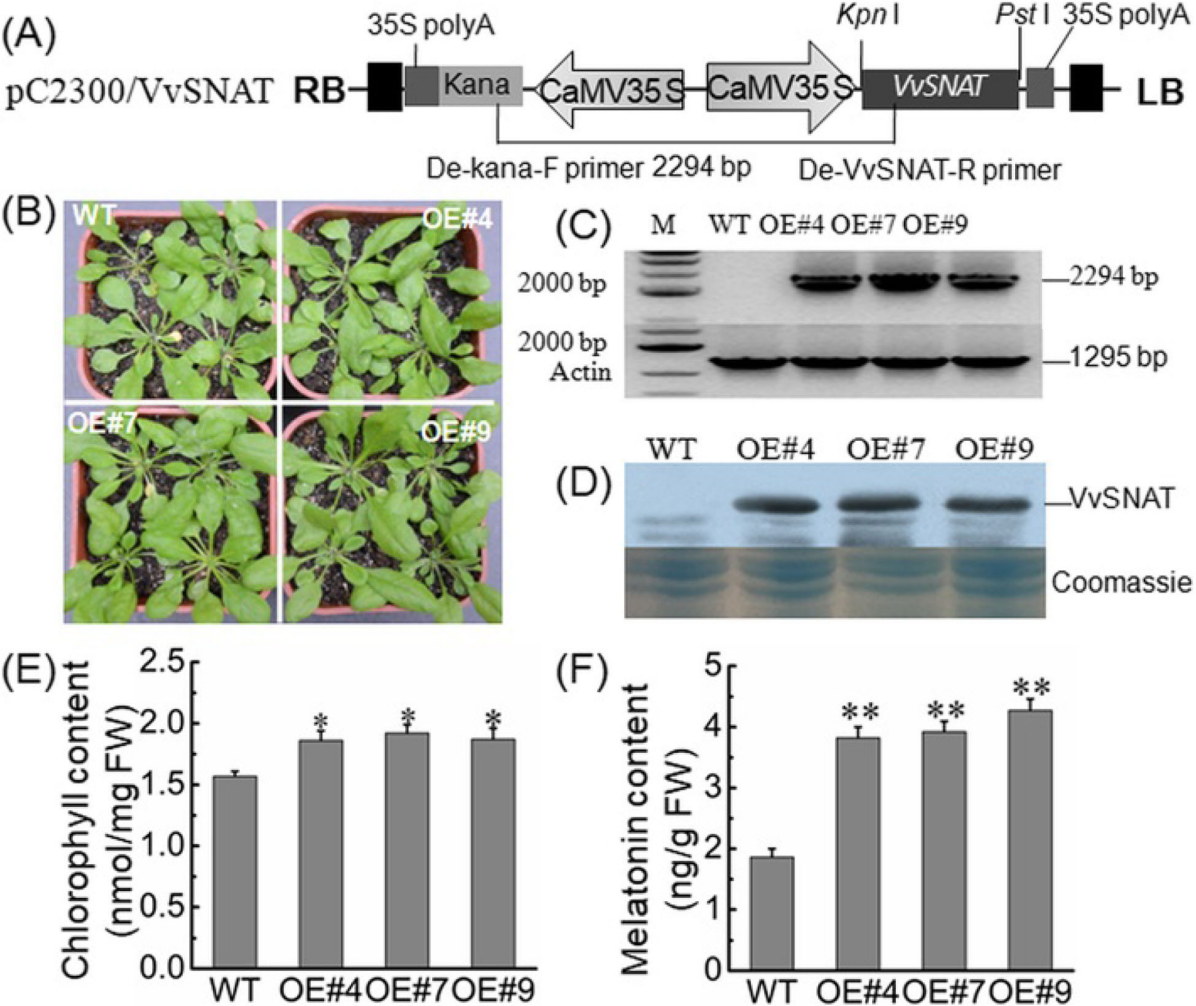
Characterization of transgenic Arabidopsis lines overexpressing *VvSNAT2*. **a** Schematic representation of the binary cassette used for the stable transformation of Arabidopsis. **b** Phenotype of transgenic and wild type (WT) plants. **c** and **d** Identification of transgenic plants using reverse transcription PCR (**c**) and western blot analysis (**d**). **e** Chlorophyll content in transgenic and WT plants. **f** Melatonin content in transgenic and WT plants. Data represent mean ± SD. Asterisks indicate statistically significant differences determined using Student’s *t*-test (*, *P* < 0.05; **, *P* < 0.01)

To identify VvSNAT2 overexpression whether promote melatonin production in transgenic plants, the *VvSNAT2* was overexpressed in Arabidopsis under the control of the CaMV *35S* promoter (Fig. 6a). Three independent homozygous transgenic lines were obtained (Fig. 6b). The T3 generation plants were checked using genomic DNA based PCR and western blot analysis (Fig. 6c, d). PCR amplification showed the presence of a specific target band comprising a fragment of *VvSNAT2*, the kanamycin selection marker and two full-length CaMV *35S* promoter sequences in the transgenic plants but not in the WT plant (Fig. 6c). Western blot analysis showed the accumulation of VvSNAT2 protein in all three independent transgenic lines of Arabidopsis (Fig. 6d). The leaves of transgenic plants were darker in color than those of WT plants. Analysis of the chlorophyll and melatonin content revealed that the three transgenic lines not only exhibited higher chlorophyll content but also contained more melatonin than the WT (Fig. 6e, f).

### Disease resistance of transgenic Arabidopsis overexpressing *VvSNAT2*

**Fig. 7:**
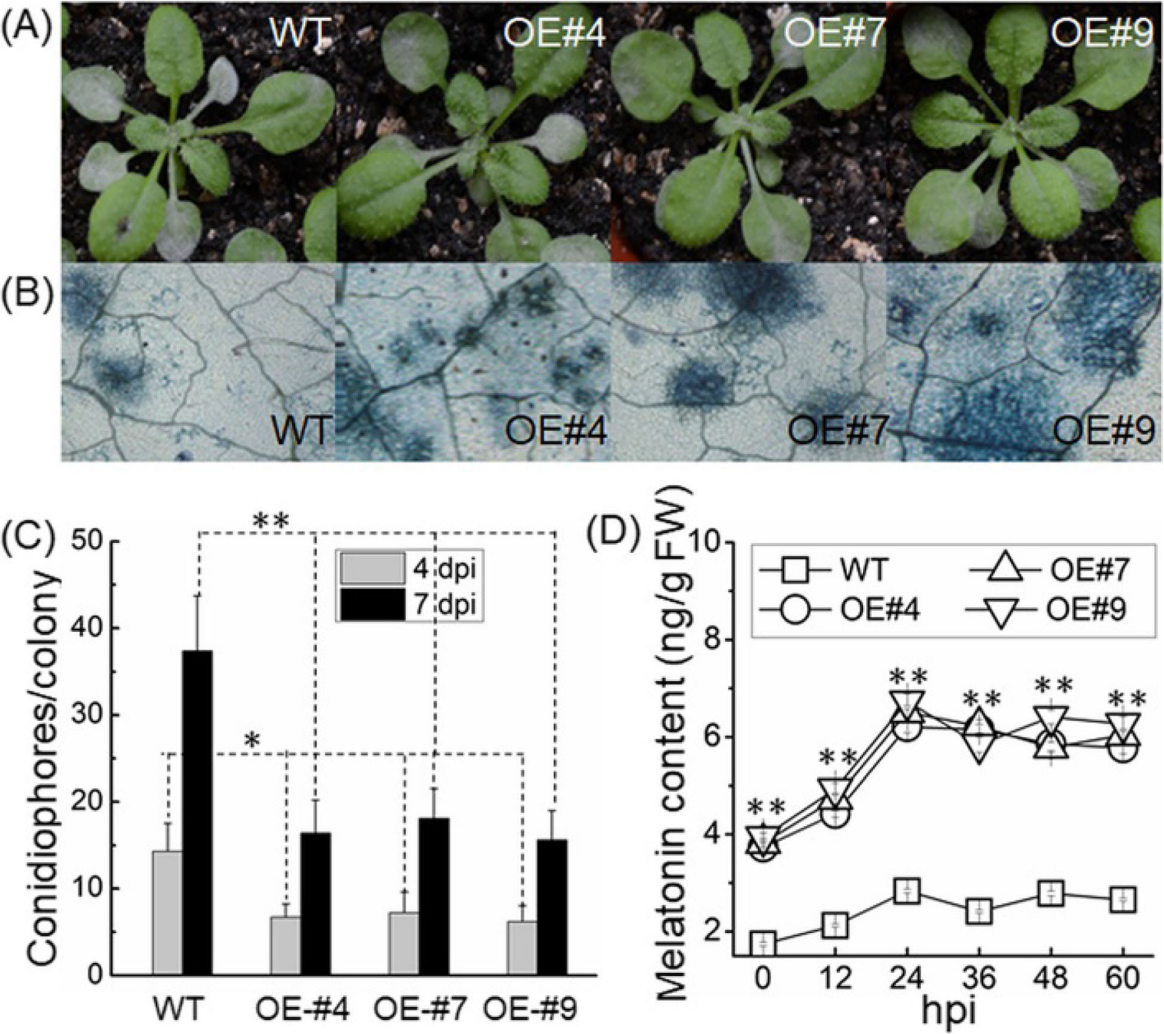
Infection of *VvSNAT2* overexpression lines of Arabidopsis with powdery mildew fungus. **a** Disease symptoms on WT and transgenic Arabidopsis plants at 7 days post-inoculation (dpi). **b** WT and transgenic Arabidopsis leaves stained with Trypan blue at 7 dpi. The results are representative of three independent experiments that yielded similar results. **c** Quantification of powdery mildew growth in Arabidopsis by calculating the number of conidiophores per colony at 4 and 7 dpi. Data represent mean ± SD of three independent biological replicates (*n* = 30). **d** Quantification of melatonin content in WT and transgenic Arabidopsis after pathogen inoculation. Data represent mean ± SD of three independent biological replicates at each time point. Asterisks indicate statistically significant differences determined using Student’s *t*-test (*, *P* < 0.05; **, *P* < 0.01)

To determine whether *VvSNAT2* is involved in defense response, transgenic Arabidopsis plants overexpressing *VvSNAT2* were challenged with the powdery mildew pathogen. The transgenic plants were more resistant to powdery mildew than WT plants (Fig. 7a). To evaluate the resistance at the histological level, infected leaves were stained with Trypan blue. Results showed more severe cell death in transgenic plants overexpressing *VvSNAT2* than in WT plants (Fig. 7b). Furthermore, to quantify fungal reproduction and development, conidiophores in transgenic and WT plants were counted. Results showed that the WT plants supported significantly more conidiophores than the transgenic plants at 7 days post-inoculation (dpi) (Fig. 7c). After challenge with powdery mildew, the melatonin content in all plants was increased. Transgenic plants exhibited peak melatonin content at 24 h post-inoculation (hpi), which was maintained until 60 hpi (Fig. 7d).

**Fig. 8:**
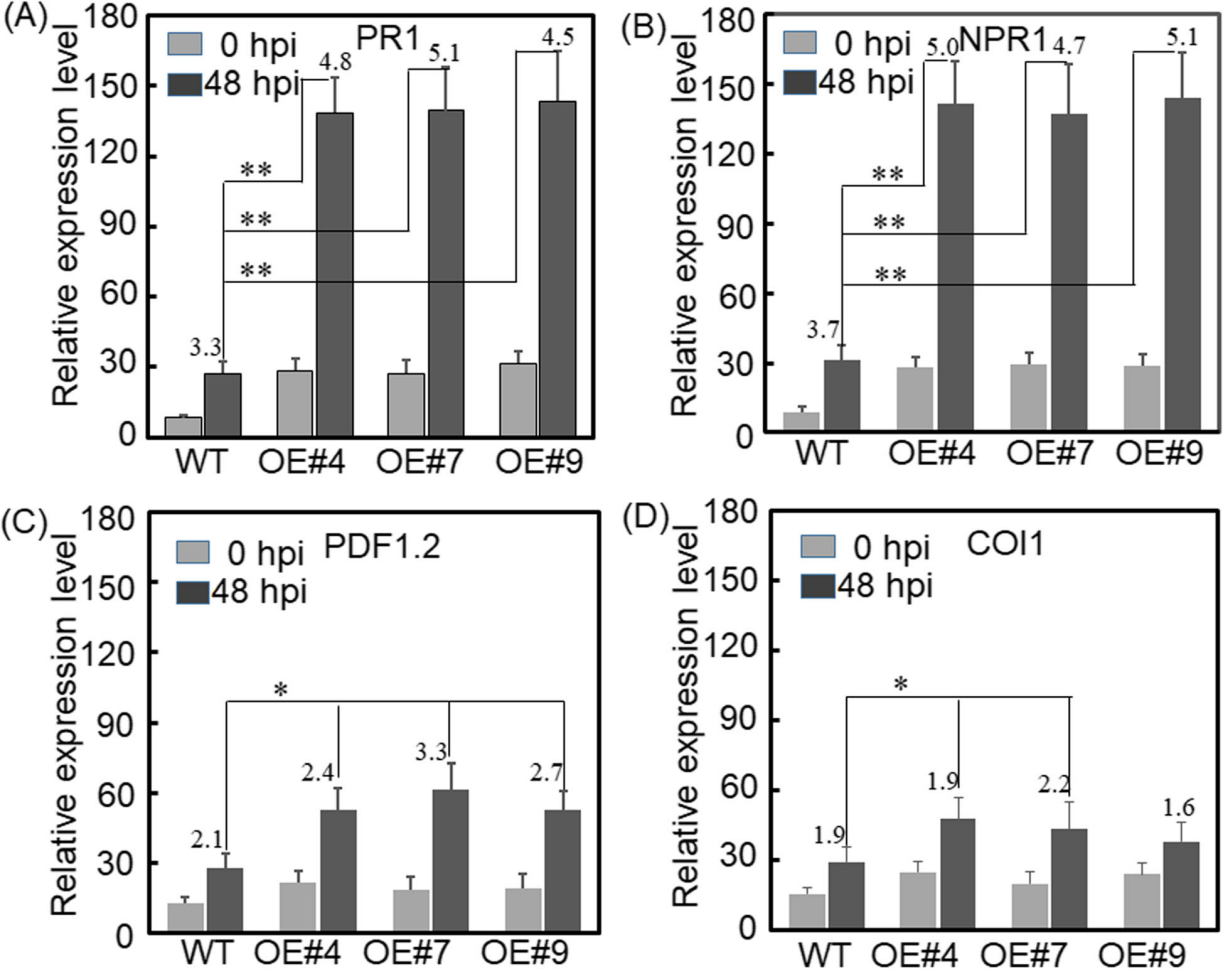
Expression of marker genes of the salicylic acid and jasmonic acid signaling pathways in WT and transgenic Arabidopsis plants. Five-week-old plants were inoculated with powdery mildew. Data represent mean ± SD of three biological replicates per genotype and time point. Asterisks indicate statistically significant differences determined using Student’s *t*-test (*, *P* < 0.05; **, *P* < 0.01). hpi, hours post-inoculation

To further examine the effects of increased melatonin production on disease resistance, the expression of genes involved in salicylic acid (SA) or jasmonic acid (JA) signaling pathways was investigated in transgenic and WT Arabidopsis plants. *PR1* and *NPR1* is the marker gene of the SA signaling pathway. In WT plants, powdery mildew infection induced the *PR1* and *NPR1* transcripts accumulation (Fig. 8a, b). In *VvSNAT2* overexpression lines, *PR1* and *NPR1* transcripts were abundant after pathogen inoculation (Fig. 8a, b). The *PR1* and *NPR1* transcripts in transgenic plants were 3.3–3.8-fold higher than those in WT plants before pathogen infection (Fig. 8a, b). At 48 hpi, the level of *PR1* and *NPR1* transcripts in transgenic plants was 5.0–5.2-fold and 4.3–4.5-fold higher, respectively, than that in WT plants (Fig. 8a, b). The marker genes of the JA signaling pathway, *PDF1.2* and *COI1*, were also induced in transgenic and WT plants after pathogen inoculation (Fig. 8c, d). However, the expression level of *PDF1.2* and *COI1* was much lower than that of *PR1* and *NPR1* in transgenic plants after pathogen inoculation (Fig. 8c, d).

## Discussion

Melatonin has been identified in many plant species, including Arabidopsis, rice, wheat, barley, corn and grapevine [4, 8, 9, 17, 21, 22]. Grapes are a highly valuable health-promoting fruit because they contain two kinds of resveratrol and are also high in melatonin [17, 20, 23]. Several studies have shown that melatonin is present in fresh berries, grape products, such as grape juice and wine, and in other plant organs, including leaves, seeds, flesh and skin [16–20, 24–26]. Although studies have been conducted to investigate the affect of genotype, developmental stage, agro-meteorological conditions and environmental factors on the melatonin content in grapevine [16, 17, 19, 20, 24], the key genes or enzymes of the melatonin biosynthetic pathway in grapevine have not yet been identified.

The *SNAT* gene belongs to the *GNAT* superfamily [8, 9], which comprises 30 members in grapevine (Table 1). Of these 30 members, only 5 contain the chloroplast transit peptide (Table 1). In rice, OsSNAT1 and OsSNAT2 have been shown to contain the chloroplast transit peptide, and both proteins are localized to the chloroplast [8, 9]. In this study, we suspected that these five *GNAT* members were candidate *SNAT* genes in grapevine. Expression in *E. coli* showed that VvGNAT16, a protein encoded by one of the five *GNAT* family members, produced a high level of *N*-acetyltryptamine using tryptamine as a substrate (Fig. 1). Additionally, phylogenetic analysis showed that VvGNAT16 grouped with OsSNAT1 and OsSNAT2 in the same subfamily (Fig. 2). Together, these results suggested that *VvGNAT16* is the *VvSNAT2* gene. Transient expression of *VvSNAT2* in Arabidopsis protoplasts and immunogold labeling of ultrathin sections of grapevine leaves showed that VvSNAT2 protein is localized in the chloroplast (Fig. 4). In rice, OsSNAT1 localizes to the chloroplast, whereas OsSNAT2 is present both in the chloroplast and cytoplasm. In Arabidopsis and *Pinus taeda*, SNAT-mCherry plasmid transformed tobacco show mCherry signal only in the chloroplasts. These results further confirm that *VvGNAT16* is the *VvSNAT2* gene.

Temperature and pH are the major factors affecting SNAT enzyme activity. Characterization of VvSNAT2 showed that it has high enzyme activity at pH 8.8 or a temperature of 45 °C (Fig. 3a, c). Compared with rice, the optimum pH of OsSNAT1 and OsSNAT2 is pH 8.8, and the maximum reaction temperature of OsSNAT2 is 45 °C [8, 9]. In Arabidopsis, the highest reaction temperature of SNAT2 is also 45 °C [27]. However, the ideal reaction temperature of SNAT2 is 55 °C in *Pinus taeda* [12]. SNAT showed high enzyme activity under 95 °C in *Synechocystis* sp. PCC 6803 and 75 °C in *Malus zumi* Mats, respectively [11, 28]. Plants have evolved a variety of responses to elevated temperatures that minimize damage and ensure protection of cellular homeostasis [29]. Plant SNAT proteins still have enzymatic activity under high tempreture conditions, which may be related to their ability to resist heat stress. It’s noted that a high level of substrate inhibition of N-acetylserotonin activity was observed. Higher level of substrate inhibition protein productivity was reported in other plants [9]. The values of *K*_m_ and *V*_max_ using serotonin were 392.5 μM and 836 pmol/min/mg protein, respectively, for VvSNAT2 (Fig. 3d). The *K*_m_ value of VvSNAT2 was similar to that of OsSNAT2 (372 μM) but different from that of OsSNAT1 (270 μM) [8, 9]. However, the *V*_max_ value of VvSNAT2 was much lower than those of OsSNAT1 and OsSNAT2 (3.3 and 4.7 nmol/min/mg protein, respectively) [8, 9]. These data indicate that the SNAT enzyme characteristics are different depending on the plant species.

The expression of VvSNAT2 protein was quickly induced in response to melatonin treatment and pathogen infection (Fig. 5), suggesting that VvSNAT2 is involved in plant defense. To test this hypothesis, we generated *VvSNAT2* overexpression Arabidopsis lines and challenged these with powdery mildew pathogen to investigate the disease resistance of transgenic plants. Results showed that VvSNAT2 expression elevated the chlorophyll and melatonin content in transgenic plants (Fig. 6). Previously, exogenous application of melatonin in Arabidopsis and *Malus domestica* has been shown to increase the chlorophyll content of leaves [27, 30, 31]. Overexpression of *VvSNAT2* in Arabidopsis resulted in the accumulation of melatonin to levels equal to that used in exogenous melatonin treatment. After pathogen infection, WT Arabidopsis plants showed more severe disease symptoms and less programmed cell death than the transgenic plants (Fig. 7a, b). Programmed cell death plays an important role in disease resistance [32]. To restrict the pathogen’s development, host plants form necrotic tissue to prevent the pathogen from assimilating nutrients [32]. Infection with pathogen also increased the melatonin level in transgenic plants (Fig. 7d). Furthermore, the expression of *PR1* and *NPR1*, marker genes of the SA signaling pathway [33], was significantly upregulated in all three transgenic lines compared with the WT 48 h after pathogen inoculation (Fig. 8a, b). Additionally, the expression of *PDF1.2* and *COI1*, marker genes of the JA signaling pathway [34], in *VvSNAT2* overexpressor lines was also higher than that in WT plants (Fig. 8c, d). However, the expression of *PDF1.2* and *COI1* was much lower than that of *PR1* and *NPR1* in the transgenic plants. These results indicate that *VvSNAT2* overexpression activates the SA and JA signaling pathways; however, the SA pathway plays a central role in VvSNAT2-mediated plant defense. The *snat* mutant of Arabidopsis exhibits decreased resistance to pathogens, reduced melatonin level and subsequently reduced SA levels during pathogen attack [21].

## Conclusions

In summary, the *VvSNAT2* gene was cloned and identified in grapevine for the first time. These results will helpful understand melatonin biosynthesis pathway in grapevine and provide basis theories for melatonin involved in plant defense. A new role of melatonin for enhancing plant defense via ER defense system was recently discovered [35]. Further work will focus on the VvSNAT2 how to response the plant defense response and the molecular mechanisms in VvSNAT2 transcriptional regulation.

## Methods

### Plant materials and treatments

Plants of the grapevine cultivar ‘Cabernet Sauvignon’ were sampled from the field. The powdery mildew fungus *Erysiphe necator* was collected from 20-year-old ‘Cabernet Sauvignon’ plants growing in the field. Transgenic and wild type (WT) plants of *Arabidopsis thaliana* ecotype Columbia (Col-0) plants were grown in vermiculite: perlite (1:1, v/v) mix in plastic pots in a growth chamber. The fungal pathogen of Arabidopsis powdery mildew, *Golovinomyces cichoracearum* (UCSC1 isolate), was maintained on Arabidopsis *phytoalexin deficient 4* (*pad4*) mutant plants. Grapevine and Arabidopsis plants were challenged with powdery mildew pathogens, as described previously [36]. Leaves of grapevine plants were sprayed with melatonin (50 mM) or double distilled water (control) at 9:00 am. The chlorophyll content of Arabidopsis leaves was measured as described previously [37]. Trypan blue was used to stain the plant leaves according to the method of Yu et al. [36]

### Identification of grapevine *GNAT* gene family and phylogenetic analysis

The latest version of the grapevine genome sequence was downloaded from the CRIBI website (http://genomes.cribi.unipd.it/). To identify the grapevine GNAT gene family, the HMM profile of the GNAT domain (Pfam00583) was used as a query to search the grapevine genome database using the Markov Model-based HMMER program. The molecular weight (MW) and isoelectric point (pI) of each protein sequence were calculated using ExPASY (http://web.expasy.org/compute_pi/). The chloroplast transit peptide sequence was predicted using the ChloroP website [38]. Amino acid sequences of OsSNAT1 and OsSNAT2 were downloaded from NCBI. Multiple sequence alignments were performed using the MEGA X software [39]. Phylogenetic analysis was performed with MEGA X using the neighbor-joining method with 1000 bootstrap replicates [39].

### RNA and genomic DNA extraction and PCR

Total RNA was extracted from grapevine and Arabidopsis leaves using the RNeasy Plant Kit (Qiagen, Beijing, China). First-strand cDNA synthesis was performed using the cDNA Synthesis Kit (TaKaRa, Dalian, China). Full-length *VvSNAT2* cDNA was amplified from grapevine RNA using PrimeSTAR® Max DNA Polymerase (TaKaRa), according to the manufacturer’s instructions, and gene-specific primers designed against the genome sequence of grapevine cultivar ‘Pinot noir’ [40]. qRT-PCR) assay was performed in a 25 μl reaction volume using SYBR Premix Ex *Taq* II kit (TaKaRa), according to the manufacturer’s instructions, and detected by a Bio-Rad IQ5 real-time PCR detection system (Bio-Rad Laboratories, Hercules, CA, USA). The *β-TUB4* gene was used as an internal control. Expression data were calculated using the 2^-ΔΔCT^ method [41]. All results were generated from three technical replicates, each of which contained three biological replicates. All primers are listed in Additional file 2: Table S1.

### Plasmid construction and plant transformation

The coding sequence of *VvSNAT2* was cloned into the pCAMBIA2300 vector under the control of the Cauliflower mosaic virus (CaMV) *35S* promoter using *Kpn*I and *Pst*I restriction sites to generate the pC2300/VvSNAT2 construct. The construct was sequenced and then transformed into *Agrobacterium tumefaciens* strain GV3101. Arabidopsis plants were stably transformed with pC2300/VvSNAT2 using the floral dip method, and PCR-positive transgenic plants were further tested for VvSNAT2 protein accumulation. Transgenic Arabidopsis plants were grown for three generations, and T3 plants were used for subsequent experiments.

### Arabidopsis protoplast isolation and transient transformation

The coding sequence of *VvSNAT2* without the termination codon was amplified using sequence-specific primers (Additional file 2: Table S1) and cloned into the pBI221-GFP vector using *Xba*I and *Kpn*I restriction sites to generate a fusion of VvSNAT2 with the green fluorescent protein (GFP). The resulting pBI221-GFP/VvSNAT2 construct was transformed into Arabidopsis protoplasts via polyethylene glycol (PEG)-mediated transformation [42]. The transformed protoplasts were cultured in the dark at 22 °C for 14 h. A Zeiss LSM 510 confocal laser scanning microscope (Zeiss, Oberkochen, Germany) was used to detect GFP fluorescence.

### Protein expression, purification and antiserum preparation

The coding sequence of *VvSNAT2* minus the chloroplast transit peptide was amplified using sequence-specific primers; this introduced *Xba*I and *Eco*RI restriction sites in the primer’s 5′-terminus. The PCR product was cloned into pGEM-T vector (TaKaRa) to generate the pGEM/VvSNAT2 plasmid, which was confirmed by sequencing. The plasmid was digested with *Xba*I and *Eco*RI restriction endonucleases to release the *VvSNAT2* fragment. The *VvSNAT2* fragment was cloned into pET-28a vector (Novagen) using *Xba*I and *Eco*RI to generate the pET/VvSNAT2 plasmid. The pET-28a and pET/VvSNAT2 plasmids were transformed into *E. coli* strain BL21 (DE3) pLysS. Protein expression in transformed *E. coli* cells was induced by the addition of 1 mM IPTG at 37 °C and examined by polyacrylamide gel electrophoresis (PAGE). The fusion protein was purified by nickel (Ni^2+^) affinity chromatography, according to the manufacturer’s instructions. Purified VvSNAT2 protein (100 μg) was mixed with complete Freund’s adjuvant, and immune New Zealand White rabbits three times at 2-week intervals. Rabbit serum was collected after injection in incomplete Freund’s adjuvant for 7 days. Antiserum was prepared as described previously [43].

### Protein extraction and western blot analysis

Total protein extract was prepared from leaves according to Wang’s method [44] and quantified using the Bradford assay. Protein extract (20 μg) was separated by SDS-PAGE, and the gels were stained with Coomassie brilliant blue. For western blot analysis, the separated proteins were transferred to PVDF membranes. The membrane was blocked with PBS buffer, and then incubated with 1000-fold dilutions of primary polyclonal antibodies for 1.5 h. The membrane was washed with PBST (Phosphate Buffered Saline with Tween 20) three times, and then incubated with goat anti-rabbit second antibody. The BCIP/NBT kits (Sigma-Aldrich, Shanghai, China) were used for monitoring the color reaction.

### Quantification of melatonin and *N*-acetylserotonin

Melatonin and *N*-acetylserotonin were identified and quantified using high performance liquid chromatography (HPLC) (Waters 2695; Waters, USA) and a fluorescence detector (Waters 2475; Waters, USA). The solid phase comprised a reverse C18 column (Kromasil 100-5C18, 250 mm × 4.6 mm, 5 μm; AkzoNobel, Sweden). Separation was performed with a linear gradient using eluent A (water with 2% formic acid) and eluent B (acetonitrile with 2% formic acid). Arabidopsis leaves (200 mg) were ground to a fine powder and extracted with 6 ml of methanol. Cell pellets were collected by centrifugation, and extracted with 300 μl of ethyl acetate. Samples were centrifuged at 13,500×*g* for 10 min. Supernatants were fractionated according to the Kang method [9]. The fraction eluted in chloroform: methanol (30:1) solution was evaporated, and the residue was dissolved in 500 μl of methanol. The solution was directly subjected to HPLC for quantification.

### Measurement of VvSNAT enzymatic activity in vitro

The enzymatic activity of VvSNAT2 was assayed according to Byeon et al. [8] For each reaction, the purified recombinant VvSNAT2 was added to 100 μl of the reaction buffer, which contained 0.5 mM of serotonin, 0.5 mM of acetyl-CoA and 100 mM of potassium phosphate (pH 8.8). The reaction was incubated at a temperature below 30 °C for 30 min. Subsequently, 25 μl of methanol was added to stop the reaction, and a 10-μl aliquot was subjected to HPLC to measure the enzymatic activity of VvSNAT2. Deferent recombinant VvSNAT2 was used with the above-described buffer to determine the substrate affinity (*K*_m_) and maximum reaction rate (*V*_max_) of VvSNAT2. The *K*_m_ and *V*_max_ values were calculated using Lineweaver-Burk plots, and the protein concentration was determined using the Bradford method [9, 45].

### Subcellular immunogold labeling and electron microscopy

Ultrathin sections of grapevine leave were prepared from Lowicryl K_4_M-embedded specimens, according to Peng’s method [46]. Ultrathin sections were transferred to a drop of 2% (w/v) bovine serum albumin (BSA) in tris-buffered saline (TBS) (pH 7.5) and blocked at room temperature for 1 h. Sections were then incubated for 1 h with the antiserum, which was diluted 100-fold in TBS buffer containing 1% (w/v) BSA. Subsequently, the sections were washed three times with TBS buffer for 10 min each. The washed sections were then placed on a drop of 10 nm gold-conjugated anti-rabbit IgG (Sigma-Aldrich) diluted 50-fold in TBS and incubated for 1 h. Samples were washed three times with TBS buffer and then two times with distilled water. Sections were then counterstained with 2% (w/v) uranyl acetate. Gold-labeled sections were examined under a JEOL (Tokyo, Japan) JEM 1220 transmission electron microscope. Rabbit pre-immune serum was used as a control.

## Supplementary information

**Additional file 1: Figure S1.** VvSNAT2 protein expression in *E. coli* and purification. 12% SDS-PAGE analysis of *E. coli* BL21(DE3) harboring pET28a vector. Lane M, pre-stained protein marker; lane 1, before IPTG induction; lane 2, after IPTG induction for 4 h cultivation; lane 3, purified VvSNAT2-His fusion protein.

**Additional file 2: Table S1.** The primers used in this study.

## Data Availability

All data generated and analyzed during this study are included in this published article. Table S1 The primers used in this study. Figure S1 VvSNAT2 protein expression in *E. coli* and purification.

## Abbreviations

ASMT: *N*-acetylserotonin methyltransferase
BSA: Bovine serum albumin
CaMV: Cauliflower mosaic virus
GFP: Green fluorescent protein
GNAT: GCN5-related *N*-acetyltransferase
hpi: Hours post-inoculation
HPLC: High performance liquid chromatography
JA: Jasmonic acid
MW: Molecular weight
ORF: Open reading frame
*pad4*: *phytoalexin deficient 4*
PAGE: Polyacrylamide gel electrophoresis
PEG: Polyethylene glycol
SA: Salicylic acid
SD: Standard deviation
SNAT: Serotonin *N*-acetyltransferase
T5H: Tryptamine5-hydroxylase
TBS: Tris-buffered saline
TDC: Tryptophan decarboxylase

## References

[1] Lerner AB, Case JD, Takahashi Y (1960). Isolation of melatonin and 5-methoxyindole-3-acetic acid from bovine pineal glands. J Biol Chem.

[2] Reiter RJ, Tan DX, Terron MP, Flores LJ, Czarnocki Z (2007). Melatonin and its metabolites: new findings regarding their production and their radical scavenging actions. Acta Biochim Pol.

[3] Reiter RJ, Melchiorri D, Sewerynek E, Poeggeler B, Barlowwalden L, Chuang J, Ortiz GG, Acunacastroviejo D (1995). A review of the evidence supporting melatonin's role as an antioxidant. J Pineal Res.

[4] Tan DX, Manchester LC, Hardeland R, Lopezburillo S, Mayo JC, Sainz RM, Reiter RJ (2003). Melatonin : a hormone, a tissue factor, an autocoid, a paracoid, and an antioxidant vitamin. J Pineal Res.

[5] Arnao MB, Hernandezruiz J (2014). Melatonin: plant growth regulator and/or biostimulator during stress?. Trends Plant Sci.

[6] Wei W, Li QT, Chu YN, Reiter RJ, Yu XM, Zhu DH, Zhang WK, Ma B, Lin Q, Zhang J (2015). Melatonin enhances plant growth and abiotic stress tolerance in soybean plants. J Exp Bot.

[7] Nawaz MA, Huang Y, Bie Z, Ahmed W, Reiter RJ, Niu M, Hameed S (2016). Melatonin: current status and future perspectives in plant science. Front Plant Sci.

[8] Byeon Y, Lee HY, Back K (2016). Cloning and characterization of the serotonin N-acetyltransferase-2 gene (SNAT2) in rice (Oryza sativa). J Pineal Res.

[9] Kang K, Lee K, Park S, Byeon Y, Back K (2013). Molecular cloning of rice serotonin N-acetyltransferase, the penultimate gene in plant melatonin biosynthesis. J Pineal Res.

[10] Lee K, Back K. Overexpression of rice serotonin N -acetyltransferase 1 in transgenic rice plants confers resistance to cadmium and senescence and increases grain yield. J Pineal Res. 2017;62(3) e12392. 10.1111/jpi.12392 28118490

[11] Byeon Y, Lee K, Park Y, Park S, Back K (2013). Molecular cloning and functional analysis of serotonin N-acetyltransferase from the cyanobacterium Synechocystis sp. PCC 6803. J Pineal Res.

[12] Park S, Byeon Y, Lee HY, Kim Y, Ahn T, Back K (2014). Cloning and characterization of a serotonin N-acetyltransferase from a gymnosperm, loblolly pine (Pinus taeda). J Pineal Res.

[13] Guo D, Zhao H, Li Q, Zhang G, Jiang J, Liu C, Yu Y (2019). Genome-wide association study of berry-related traits in grape [Vitis vinifera L.] based on genotyping-by-sequencing markers. Hortic Res.

[14] Yu Y, Jiao Z, Bian L, Wan Y, Yu K, Zhang G, Guo D (2019). Overexpression of Vitis vinifera VvbZIP60 enhances Arabidopsis resistance to powdery mildew via the salicylic acid signaling pathway. Sci Hortic.

[15] Xu W, Yu Y, Zhou Q, Ding J, Dai L, Xie X, Xu Y, Zhang C, Wang Y (2011). Expression pattern, genomic structure, and promoter analysis of the gene encoding stilbene synthase from Chinese wild Vitis pseudoreticulata. J Exp Bot.

[16] Meng J, Xu T, Wang Z, Fang Y, Xi Z, Zhang Z (2014). The ameliorative effects of exogenous melatonin on grape cuttings under water-deficient stress: antioxidant metabolites, leaf anatomy, and chloroplast morphology. J Pineal Res.

[17] Iriti M, Varoni EM (2016). The good health of Bacchus: melatonin in grapes, the unveiled myth. LWT- Food Sci Technol.

[18] Gomez FJV, Raba J, Cerutti S, Silva MF (2012). Monitoring melatonin and its isomer in Vitis vinifera cv. Malbec by UHPLC-MS/MS from grape to bottle. J Pineal Res.

[19] Boccalandro HE, Gonzalez CV, Wunderlin DA, Silva MF (2011). Melatonin levels, determined by LC-ESI-MS/MS, fluctuate during the day/night cycle in Vitis vinifera cv Malbec: evidence of its antioxidant role in fruits. J Pineal Res.

[20] Meng J, Xu T, Song C, Yu Y, Hu F, Zhang L, Zhang Z, Xi Z (2015). Melatonin treatment of pre-veraison grape berries to increase size and synchronicity of berries and modify wine aroma components. Food Chem.

[21] Lee HY, Byeon Y, Tan DX, Reiter RJ, Back K (2015). Arabidopsis serotonin N-acetyltransferase knockout mutant plants exhibit decreased melatonin and salicylic acid levels resulting in susceptibility to an avirulent pathogen. J Pineal Res.

[22] Arnao MB, Hernandezruiz J (2015). Functions of melatonin in plants: a review. J Pineal Res.

[23] Jang M, Cai L, Udeani GO, Slowing K, Thomas CF, Beecher CWW, Fong HHS, Farnsworth NR, Kinghorn AD, Mehta RG (1997). Cancer chemopreventive activity of resveratrol, a natural product derived from grapes. Science.

[24] Meng J, Shi T, Song S, Zhang Z, Fang Y (2017). Melatonin in grapes and grape-related foodstuffs: a review. Food Chem.

[25] Vitalini S, Gardana C, Simonetti P, Fico G, Iriti M (2013). Melatonin, melatonin isomers and stilbenes in Italian traditional grape products and their antiradical capacity. J Pineal Res.

[26] Vitalini S, Gardana C, Zanzotto A, Simonetti P, Faoro F, Fico G, Iriti M (2011). The presence of melatonin in grapevine (*Vitis vinifera* L.) berry tissues. J Pineal Res.

[27] Byeon Y, Choi G, Lee HY, Back K (2015). Melatonin biosynthesis requires N-acetylserotonin methyltransferase activity of caffeic acid O-methyltransferase in rice. J Exp Bot.

[28] Wang L, Feng C, Zheng X, Guo Y, Zhou F, Shan D, Liu X, Kong J (2017). Plant mitochondria synthesize melatonin and enhance the tolerance of plants to drought stress. J Pineal Res.

[29] Ohama N, Sato H, Shinozaki K, Yamaguchi-Shinozaki K (2017). Transcriptional regulatory network of plant heat stress response. Trends Plant Sci.

[30] Wang P, Yin L, Liang D, Li C, Ma F, Yue Z (2012). Delayed senescence of apple leaves by exogenous melatonin treatment: toward regulating the ascorbate–glutathione cycle. J Pineal Res.

[31] Wang P, Sun X, Li C, Wei Z, Liang D, Ma F (2013). Long-term exogenous application of melatonin delays drought-induced leaf senescence in apple. J Pineal Res.

[32] Mittler R (2017). ROS are good. Trends Plant Sci.

[33] Thomma BPHJ, Eggermont K, Penninckx IAMA, Mauchmani B, Vogelsang R, Cammue BPA, Broekaert WF (1998). Separate jasmonate-dependent and salicylate-dependent defense-response pathways in Arabidopsis are essential for resistance to distinct microbial pathogens. Proc Natl Acad Sci U S A.

[34] De Vos M, Van Oosten VR, Van Poecke RMP, Van Pelt JA, Pozo MJ, Mueller MJ, Buchala AJ, Metraux J, Van Loon LC, Dicke M (2005). Signal signature and transcriptome changes of Arabidopsis during pathogen and insect attack. Mol Plant-Microbe Interact.

[35] Lee HY, Back K (2018). Melatonin plays a pivotal role in conferring tolerance against endoplasmic reticulum stress via mitogen-activated protein kinases and bZIP60 in *Arabidopsis thaliana*. Melatonin Res.

[36] Yu Y, Xu W, Wang J, Wang L, Yao W, Yang Y, Xu Y, Ma F, Du Y, Wang Y (2013). The Chinese wild grapevine (Vitis pseudoreticulata) E3 ubiquitin ligase Erysiphe necator-induced RING finger protein 1 (EIRP1) activates plant defense responses by inducing proteolysis of the VpWRKY11 transcription factor. New Phytol.

[37] Kanwischer M, Porfirova S, Bergmuller E, Dormann P (2005). Alterations in tocopherol cyclase activity in transgenic and mutant plants of Arabidopsis affect tocopherol content, tocopherol composition, and oxidative stress. Plant Physiol.

[38] Emanuelsson O, Nielsen H, Von Heijne G (1999). ChloroP, a neural network-based method for predicting chloroplast transit peptides and their cleavage sites. Protein Sci.

[39] Kumar S, Stecher G, Li M, Knyaz C, Tamura K, Battistuzzi FU (2018). MEGA X: molecular evolutionary genetics analysis across computing platforms. Mol Biol Evol.

[40] Jaillon O, Aury J, Noel B, Policriti A, Clepet C, Casagrande A, Choisne N, Aubourg S, Vitulo N, Jubin C (2007). The grapevine genome sequence suggests ancestral hexaploidization in major angiosperm phyla. Nature.

[41] Livak KJ, Schmittgen TD (2001). Analysis of relative gene expression data using real-time quantitative PCR and the 2(−Delta Delta C(T)) method. Methods.

[42] Yoo SD, Cho YH, Sheen J (2007). Arabidopsis mesophyll protoplasts: a versatile cell system for transient gene expression analysis. Nat Protoc.

[43] Palmer HM (2000). Using antibodies: a laboratory manual. J Antimicrob Chemother.

[44] Wang W, Vignani R, Scali M, Cresti M (2006). A universal and rapid protocol for protein extraction from recalcitrant plant tissues for proteomic analysis. Electrophoresis.

[45] Bradford MM (1976). A rapid and sensitive method for the quantitation of microgram quantities of protein utilizing the principle of protein-dye binding. Anal Biochem.

[46] Peng YB, Lu Y, Zhang D (2003). Abscisic acid activates ATPase in developing apple fruit especially in fruit phloem cells. Plant Cell Environ.

