# Supplementary material for: Molecular cloning and characterization of a grapevine (Vitis vinifera L.) serotonin N-acetyltransferase (VvSNAT2) gene involved in plant defense
Source: BMC Genomics. 2019 Nov 20;20:880. doi: 10.1186/s12864-019-6085-3 (PMC6868852; doi:10.1186/s12864-019-6085-3)
Supplement: Supplementary file 1 — Additional file 1: Figure S1. VvSNAT2 protein expression in E. coli and purification. 12% SDS-PAGE analysis of E. coli BL21(DE3) harboring pET28a vector. Lane M, pre-stained protein marker; lane 1, before IPTG induction; lane 2, after IPTG induction for 4 h cultivation; lane 3, purified VvSNAT2-His fusion protein. [file 12864_2019_6085_MOESM1_ESM.docx]

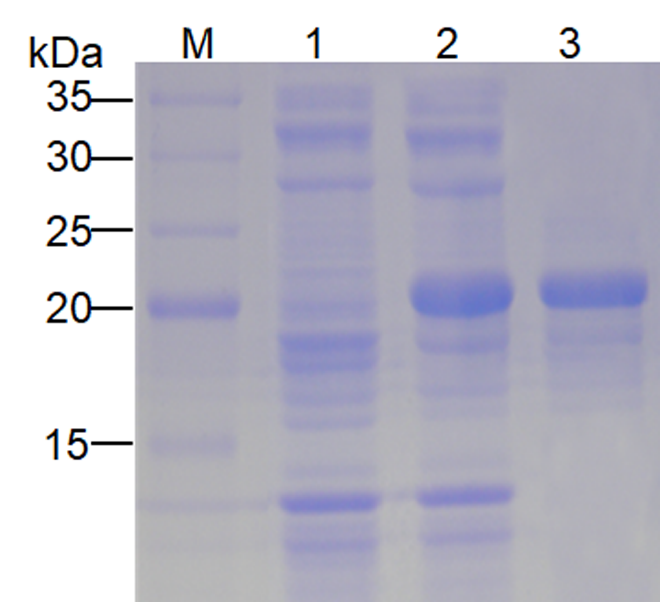


Supplementary Figure 1 VvSNAT2 protein expression in *E. coli* and purification.

12% SDS-PAGE analysis of *E. coli* BL21(DE3) harboring pET28a vector. Lane M, pre-stained protein marker; lane 1, before IPTG induction; lane 2, after IPTG induction for 4 h cultivation; lane 3, purified VvSNAT2-His fusion protein.
