# Supplementary material for: Molecular cloning and characterization of a grapevine (Vitis vinifera L.) serotonin N-acetyltransferase (VvSNAT2) gene involved in plant defense
Source: BMC Genomics. 2019 Nov 20;20:880. doi: 10.1186/s12864-019-6085-3 (PMC6868852; doi:10.1186/s12864-019-6085-3)
Supplement: Supplementary file 2 — Additional file 2: Table S1. The primers used in this study. [file 12864_2019_6085_MOESM2_ESM.docx]

Supplementary Table 1 The primers used in this study.

| Primer name | Primer sequence(5’-3’) | Purpose |
| --- | --- | --- |
| VvGNAT10-F  VvGNAT10-R | GGATCCATCGCCAACTCTTCTGATGTTCCC  CTCGAGACTAAAACTTTCGTAAGCTTGAAGAGC | Expression in *E. coli*. |
| VvGNAT15-F  VvGNAT15-R  VvGNAT16-F  VvGNAT16-R  VvGNAT17-F  VvGNAT17-R  SubVvSNAT-F  SubVvSNAT-R | GGATCCGCGGCGGTGGAAGAGGATG  CTCGAGGATGGATCTCCTGAAGCAAGAGC  GGATCCGCTTCCGCCCGGCCCTCCCA  CTCGAGCTTGTTTCGCTTCCTGGAATACACC  GGATCCGCCGAAACTGTATCCGAGGACCA  CTCGAGCACATCATAGTTGACAGGGAGACC  GGTACCATGTTGCTACGTGGCATCGTAT  TCTAGAAAATTTATTGTTTCGCTTCCTGG | Expression in *E. coli*.  Expression in *E. coli*.  Expression in *E. coli*.  Sublocalization |
| De-Kana-F  De- VvSANT-R | AGCAAGGTGAGATGACAGGAGA  GAGAGGGTTGAGGGTAGAGGG | Identification transgenic |
| qAtPR1-F  qAtPR1-R  qAtNPR1-F  qAtNPR1-R  qAtPDF1.2-F  qAtPDF1.2-R  qAtCOI-F  qAtCOI-R  β-TUB 4-F  β-TUB 4-R | GGAGCTACGCAGAACAACTAAGA  CCCACGAGGATCATAGTTGCAACTGA  GCTCTGCTCGTCAATGGTTATC  GAGGAGTCGGTGTTATCGGTAG  TCATGGCTAAGTTTGCTTCC  AATACACACGATTTAGCACC  GTGTCCTAATTTGGAAGTTCTCG  CTCCATTCCTTGTTCATCTGC  GAGGGAGCCATTGACAACATCTT  GCGAACAGTTCACAGCTATGTTCA | qRT-PCR  qRT-PCR  qRT-PCR  qRT-PCR  qRT-PCR |
